# Perinatal depression and psychiatric comorbidities during the life course: a Swedish nationwide register based study

**DOI:** 10.1192/j.eurpsy.2025.571

**Published:** 2025-08-26

**Authors:** E. Bränn, D. Lu, A. Duna

**Affiliations:** 1 Institute of Environmental Medicine, Karolinska Institutet, Stockholm, Sweden

## Abstract

**Introduction:**

Women with a history of major depression are at risk of perinatal depression (PND). The associations between PND and other types of psychiatric disorders are less clear, although a recent GWAS revealed genetic correlations with almost all psychiatric disorders.

**Objectives:**

The aim of this study was to examine the association between PND and overall, and 17 type-specific, psychiatric disorders in a life course approach.

**Methods:**

Leveraging Swedish nationwide health register and primary care data, we included all birthing women diagnosed with depression or prescribed for antidepressants during pregnancy or within a year postpartum, i.e., women with PND (n=122,720), during 2001-2022. Using incidence density sampling, we matched each case to 10 unaffected birthing women. We ascertained any diagnosis of psychiatric disorder over the lifetime from the National Patient Register. Using multivariable conditional logistic regressions, we estimated the association between PND and any, or subtypes of, psychiatric disorders dated before or after the PND diagnosis in a liftecourse approach. Moreover, we conducted a nested case control study to investigate the association of psychiatric disorders and subsequent PND, and a matched cohort study to investigate PND and subsequent psychiatric disorders.

**Results:**

In preliminary results, at a mean age of 31.0, we found that PND was highly associated with any other psychiatric disorders (adjusted odds ratio (aOR)=9.02, 95%CI 8.9-9.2). The association remained when excluding depression (aOR=6.2, 95%CI 6.1-6.3), and was comparable for psychiatric disorders dated before PND diagnosis (aOR = 9.0), whereas attenuated for diagnoses dated after PND (OR= 3.9). Most pronounced association was noted for bipolar, personality disorders, depression, and anxiety. The association was stronger in primiparous women and in women born ourside of Europe (p-for interaction <0.001).

**Conclusions:**

Throughout life course, PND is associated with psychiatric disorders, particularly with bipolar disorder, personality disorder, depression and anxiety. These findings may shed light on shared genetic/risk factors between PND and other psychiatric disorders.

**Disclosure of Interest:**

None Declared

